# Triglyceride-glucose index predicts sepsis-associated acute kidney injury and length of stay in sepsis: A MIMIC-IV cohort study

**DOI:** 10.1016/j.heliyon.2024.e29257

**Published:** 2024-04-04

**Authors:** Yijiao Fang, Bo Xiong, Xue Shang, Fan Yang, Yuehao Yin, Zhirong Sun, Xin Wu, Jun Zhang, Yi Liu

**Affiliations:** Department of Anesthesiology, Shanghai Cancer Centre, Fudan University, Shanghai, 200032, China

**Keywords:** Triglyceride-glucose index, Insulin resistance, Sepsis, Sepsis-associated acute kidney injury, Length of stay, MIMIC-IV database

## Abstract

**Background:**

Inflammation and stress response may be related to the occurrence of sepsis-associated acute kidney injury (SA-AKI) in patients with sepsis.Insulin resistance (IR) is closely related to the stress response, inflammatory response, immune response and severity of critical diseases. We assume that the triglyceride-glucose (TyG) index, an alternative indicator for IR, is associated with the occurrence of SA-AKI in patients with sepsis.

**Methods:**

Data were obtained from The Medical Information Mart for Intensive Care-IV(MIMIC-IV) database in this retrospective cohort study. Univariate and multivariate logistic regression analysis and multivariate restricted cubic spline(RCS) regression were conducted to evaluate the association between TyG index and SA-AKI, length of stay (LOS). Subgroup and sensitivity analyses were performed to verify the robustness of the results.

**Results:**

The study ultimately included data from 1426 patients with sepsis, predominantly of white ethnicity (59.2%) and male sex (56.4%), with an SA-AKI incidence rate of 78.5%. A significant linear association was observed between the TyG index and SA-AKI (OR, 1.40; 95% confidence interval(CI) [1.14–1.73]). Additionally, the TyG index demonstrated a significant correlation with the length of stay (LOS) in both the hospital (β, 1.79; 95% CI [0.80–2.77]) and the intensive care unit (ICU) (β, 1.30; 95% CI [0.80–1.79]). Subgroup and sensitivity analyses confirmed the robustness of these associations.

**Conclusion:**

This study revealed a strong association between the TyG index and both SA-AKI and length of stay in patients with sepsis. These findings suggest that the TyG index is a potential predictor of SA-AKI and the length of hospitalization in sepsis cases, broadening its application in this context. However, further research is required to confirm whether interventions targeting the TyG index can genuinely enhance the clinical outcomes of patients with sepsis.

## Key points

1. After adjusting for all clinically relevant covariates, a high TyG index as a continuous variable or quartile categorical variable was significantly associated with a high incidence of SA-AKI.
2. In sepsis patients, a high TyG index is significantly associated with longer LOS.
3. The subgroup analysis results are consistent with the main results mentioned above, and the TyG index may have a more significant predictive value for LOS in adult sepsis patients<65 years old (LOS in the hospital, P for interaction = 0.029; LOS in the ICU, P for interaction = 0.035).
4. It is worth noting that we have adjusted the use of various drugs that may affect outcomes, including high-risk nephrotoxins, diuretics, glucocorticoids and vasopressors (specific medication types are detailed in Table S5).

After separately excluding non-White ethnic groups, patients using high-risk nephrotoxins, and patients with concurrent CKD, the association between the TyG index and the incidence of SA-AKI and LOS remained stable.

## 1 Introduction

Sepsis is a systemic inflammatory response syndrome caused by infection, and is characterized by a dysregulated host response to infection, leading to life-threatening organ dysfunction [1].Sepsis is a major contributor to mortality and critical illness worldwide, with an increasing incidence trend [[2], [3], [4], [5]].

The kidneys are among the organs most affected by sepsis, with reports indicating that 45–70% of cases of acute kidney injury(AKI) cases are attributed to sepsis [[6], [7], [8], [9]]. SA-AKI, characterized by various clinical phenotypes, is closely linked to an elevated risk of adverse outcomes including prolonged hospitalization, cardiovascular events, and mortality, potentially indicating a poorer prognosis than typical AKI cases [[10], [11], [12], [13], [14]]. The early identification of patients at risk of SA-AKI and prompt implementation of appropriate interventions are of paramount importance to mitigate further renal damage.

Diagnosing AKI in septic patients can be challenging due to reduced muscle perfusion, decreased creatinine production, a gradual rise in serum creatinine levels, and concurrent use of diuretics and fluid resuscitation, which limits the applicability of serum creatinine or urine output for early SA-AKI detection [12,14,15]. Moreover, owing to the compensatory function of the kidneys, significant increases in serum creatinine and urine output are often observed after severe renal damage has occurred, potentially leading to a missed window for optimal intervention. Consequently, the exploration of novel biomarkers associated with SA-AKI holds promise for the early diagnosis of this condition, and represents an area that requires further in-depth research [16].

Earlier studies have emphasized that infection and inflammation lead to significant changes in lipids and lipoproteins, resulting in lower levels of these biomolecules in patients with sepsis [17,18]. Several studies have investigated the potential of lipoproteins, particularly high-density lipoprotein (HDL), as predictive biomarkers and therapeutic targets for sepsis and kidney injury, given their ability to correct dyslipidemia and modulate the inflammatory cascade response [19,20]. Recent studies indicate a diminished risk of stage 2 or 3 AKI and shorter ICU stays in septic patients treated with the HDL-mimetic drug CER-001 [21]. Further investigation into the involvement of lipoproteins and lipids in sepsis development and their impact on renal injury is warranted.

The TyG index, which serves as an alternative marker for IR, is associated with pathological conditions such as systemic inflammation, endothelial dysfunction, and oxidative stress [22,23].Prior research has demonstrated an association between TyG index and contrast-induced acute kidney injury [24]. However, the relationship between the TyG index and SA-AKI in septic patients remains unknown; therefore, we assessed the relationship between TyG index and SA-AKI in septic patients and further evaluated the association between TyG index and LOS in this study.

## 2 Methods

### 2.1 Study design and data sources

This was a retrospective cohort study. Data for this study were extracted from an electronic health record dataset, the MIMIC-IV database (version 2.2), which encompasses intensive care data from over 70,000 patients admitted to the Beth Israel Deaconess Medical Center (BIDMC) intensive care units between 2008 and 2019 [25]. In accordance with the Health Insurance Portability and Accountability Act (HIPAA) Safe Harbor provision, patient identifiers within the MIMIC-IV database were removed, and one author (Yi Liu) obtained permission to access the dataset(certification number 52949197). The database was approved by the review committee of the Massachusetts Institute of Technology and Beth Israel Deaconess Medical Center, and the requirement for informed consent was waived. All reports adhered to the Strengthening the Reporting of Observational Studies in Epidemiology (STROBE) guidelines [26].

**Fig. 1:**
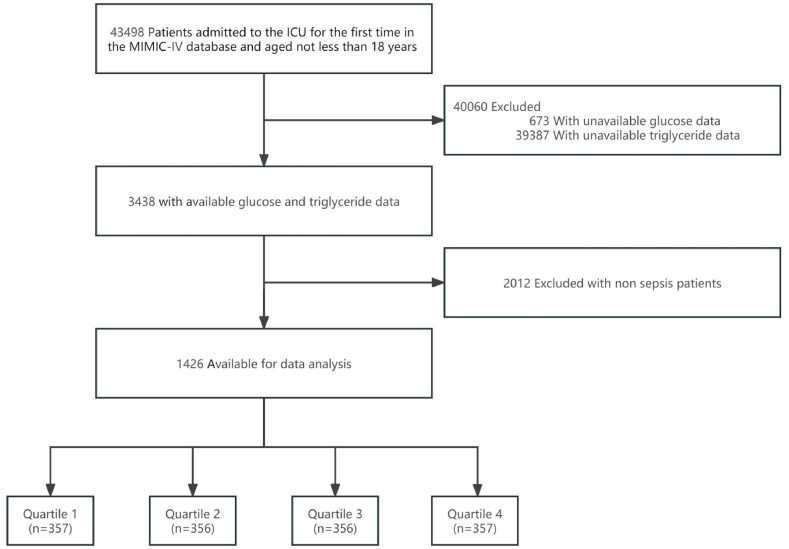
Flow chart of patients selection for analytic.

This study involved the selection of 43,498 adult patients (aged ≥18 years) from the MIMIC-IV database who were admitted to the ICU for the first time. After excluding patients with missing blood glucose data (n = 673), missing triglyceride data (n = 39,387), and those without a diagnosis of sepsis (n = 2012), data from a total of 1426 patients were included in the analysis. Patients were stratified into four groups based on TyG index quartiles (Fig. 1).

### 2.2 Ethics

Ethical approval for The MIMIC database by the institutional review boards of the Beth Israel Deaconess Medical Center (2001-P-001699/14) and the Massachusetts Institute of Technology (No. 0403000206), which waived the requirement for individual patient consent and approved the data sharing initiative. The data in the MIMIC-IV database is publicly available, therefore, the ethical approval statement and the requirement for informed consent were waived for this study.

### 2.3 Data collection and definitions

We utilized structured query language to extract clinically relevant data from the MIMIC-IV database. The collected data included demographic information (age, sex, insurance, race), comorbidities (chronic kidney disease (CKD), chronic pulmonary disease, liver disease, diabetes, malignant cancer, congestive heart failure (CHF), myocardial infarction (MI)), severity scores (sequential organ failure assessment (SOFA), Charlson comorbidity index (CCI), simplified acute physiology score II (SAPS II)), and laboratory parameters (serum creatinine (SCr), estimated glomerular filtration rate (eGFR), TyG index, and hemoglobin). Data related to the use of high-risk nephrotoxins, vasopressors medications, glucocorticoids, diuretics, acetaminophen, and albumin 5%/20% during the first two days of ICU admission were also extracted. Specific types of these medications are listed in Table S5. Additionally, data on mechanical ventilation for ≥24 h within the first two days of ICU admission were collected. In this cohort study, all screened variables had missing values of less than 1%; therefore, interpolation methods were not used.

In this study, the diagnosis of sepsis was based on Sepsis-3 criteria [1]. Organ dysfunction in this study was defined as an increase of two or more points in the Sequential Assessment of Organ Failure (SOFA) score. Infections and other comorbidities were defined using the International Classification of Diseases and Ninth Revision (ICD-9) and the International Classification of Diseases and Tenth Revision (ICD-10) codes for diagnosis. The estimated glomerular filtration rate (eGFR) was calculated using the 2009 CKD Epidemiology Collaboration (CKD-EPI) creatinine equation [27]. The TyG index was computed using the following formula: ln [fasting TG (mg/dl) × fasting glucose (mg/dl)]/2 [28].

### 2.4 Outcomes

The primary outcome, SA-AKI, was defined as the occurrence of AKI within 7 days of ICU admission in septic patients [29]. In accordance with the Kidney Disease: Improving Global Outcomes (KDIGO) guidelines, AKI was defined as a serum creatinine (SCr) level that increased by ≥ 0.3 mg/dL above baseline within 48 h or urinary output less than <0.5 mL/kg/h for 6 h [30].In this study, SCr levels were used to diagnose AKI in stages 1, 2, and 3. Urine output was also used for diagnosis when the interval between the two SCr records was less than 6 h. Secondary outcomes included the length of hospital stay and ICU admission time. Patients who died within 7 days of ICU admission without developing any stage of AKI were considered to have no SA-AKI.

### 2.5 Statistical analyses

Data analysis was conducted using the statistical software package R (http://www.R-project.org, R Foundation) and Free Statistics software (version 1.8). Continuous variables are presented as mean ± standard deviation or median (interquartile range), and differences in continuous variables were assessed using analysis of variance or the Mann-Whitney *U* test. Categorical variables are presented as frequencies and percentages, and the chi-squared test or Fisher's exact test was used for categorical variable analysis.

Multivariable logistic regression analysis was performed to assess the correlation between the TyG index (both as a continuous variable and categorized by quartiles) and the risk of SA-AKI. To evaluate the relationship between the TyG index and LOS, multiple linear regression analysis was conducted, and the B coefficients and 95% confidence intervals (CIs) were calculated. Clinical covariates for adjustment were determined based on the results of univariate logistic regression analysis, clinical expertise, and previous relevant clinical studies.Model 1 adjusted for age, gender, race, and insurance status.Model 2, built upon Model 1, further adjusted for comorbidities including liver disease,CKD, CHF, MI, diabetes, and malignant cancer. Model 3, extending Model 2, additionally adjusted for CCI, eGFR, SOFA score, SAPS II, serum creatinine, and hemoglobin.Model 4, encompassing all covariates from Model 3, also included adjustments for the use of vasopressors medications, high-risk nephrotoxins, glucocorticoids, diuretics, acetaminophen, albumin 5%/20%, and mechanical ventilation time ≥24 h.

We employed a multivariate RCS model to ascertain the linear relationship between TyG index and SA-AKI. The TyG index was used as a continuous variable in the model (with the lowest quartile of TyG index values serving as the reference group). After adjusting for covariates (Model 4), p-values for trends were obtained by using quartile levels as ordinal variables.

Subgroup and interaction analyses were performed using multivariate logistic regression and multiple linear regression, respectively. These analyses included covariate adjustments that were consistent with those in Model 4. The specific subgroups analyzed were determined by age, sex, diabetes, CKD, SOFA score, SAPS II score, and the use of high-risk nephrotoxins. These analyses were performed to validate the robustness of TyG index in predicting SA-AKI and LOS across different subgroups.

Several sensitivity analyses were conducted to further assess the robustness of our study results. First, considering the potential correlation between medication therapy, AKI and LOS, we performed an analysis after excluding the patients who used high-risk nephrotoxins. Second, as the majority of patients in our study cohort were of White ethnicity and considering the impact of racial differences on outcomes, we conducted a sensitivity analysis by excluding non-White patients. Third, the presence of concomitant CKD may have a potential impact on the occurrence of AKI and LOS. To validate our results, we conducted a sensitivity analysis by excluding patients with concomitant CKD. In all tests, a significance level of P < 0.05 was considered statistically significant.

## 3 Results

The final study cohort comprised 1426 patients diagnosed with sepsis from the MIMIC-IV database. The proportion of missing data for the relevant variables in the study population was less than 1% (Table S4). The included patients were predominantly white (59.2%) and male (56.4%), with a 78.5% incidence of SA-AKI within seven days of ICU admission. The median length of hospital stay was 11.1 days (IQR, 6.2 to 18.8), and the median ICU stay was 5.0 days (IQR, 2.7 to 9.8). The average age of the study population was 62.1 years (SD 17.5) (Table 1).

**Table 1:** Baseline characteristics and outcomes of sepsis patients grouped according to TyG index quartiles.

Categories	Overall(N = 1426)	Q1 (N = 357)	Q2 (n = 356)	Q3 (n = 356)	Q4 (n = 357)	*P*-value
**Demographic**						
Age,year, Mean ± SD	62.1 ± 17.5	65.8 ± 18.9	64.6 ± 16.6	61.1 ± 16.9	57.0 ± 16.0	<0.001
Sex, n (%)						0.084
Male	804 (56.4)	183 (51.3)	198 (55.6)	208 (58.4)	215 (60.2)	
Female	622 (43.6)	174 (48.7)	158 (44.4)	148 (41.6)	142 (39.8)	
Insurance, n (%)						0.028
Medicaid	120 (8.4)	28 (7.8)	27 (7.6)	35 (9.8)	30 (8.4)	
Medicare	588 (41.2)	165 (46.2)	150 (42.1)	152 (42.7)	121 (33.9)	
Other	718 (50.4)	164 (45.9)	179 (50.3)	169 (47.5)	206 (57.7)	
Race, n (%)						0.225
White	844 (59.2)	216 (60.5)	216 (60.7)	202 (56.7)	210 (58.8)	
African American	116 (8.1)	36 (10.1)	29 (8.1)	27 (7.6)	24 (6.7)	
Hispanic or Latino	40 (2.8)	7 (2)	9 (2.5)	17 (4.8)	7 (2)	
Asian	34 (2.4)	6 (1.7)	8 (2.2)	13 (3.7)	7 (2)	
Unknown or other	392 (27.5)	92 (25.8)	94 (26.4)	97 (27.2)	109 (30.5)	
**Comorbidities**						
CKD,n (%)	255 (17.9)	51 (14.3)	77 (21.6)	61 (17.1)	66 (18.5)	0.080
Chronic pulmonary disease,n (%)	338 (23.7)	78 (21.8)	87 (24.4)	86 (24.2)	87 (24.4)	0.822
Liver disease,n (%)	285 (20.0)	64 (17.9)	56 (15.7)	85 (23.9)	80 (22.4)	0.022
Diabetes,n (%)	408 (28.6)	44 (12.3)	82 (23)	117 (32.9)	165 (46.2)	<0.001
Malignant cancer,n (%)	142 (10.0)	32 (9)	34 (9.6)	37 (10.4)	39 (10.9)	0.824
CHF,n (%)	411 (28.8)	95 (26.6)	105 (29.5)	116 (32.6)	95 (26.6)	0.237
MI,n (%)	302 (21.2)	61 (17.1)	78 (21.9)	90 (25.3)	73 (20.4)	0.060
**Severity scores**						
SOFA, Median (IQR)	3.0 (2.0, 4.0)	3.0 (2.0, 4.0)	3.0 (2.0, 4.0)	3.0 (2.0, 5.0)	4.0 (2.0, 6.0)	<0.001
CCI, Median (IQR)	6.0 (4.0, 8.0)	6.0 (4.0, 8.0)	6.0 (4.0, 8.0)	6.0 (4.0, 8.0)	5.0 (3.0, 7.0)	0.012
SAPS II, Mean ± SD	40.8 ± 15.1	38.8 ± 13.2	39.2 ± 14.4	39.9 ± 14.9	45.2 ± 16.8	<0.001
**Laboratory parameters**						
Serum creatinine,mg/dL,Median (IQR)	1.1 (0.8, 1.8)	0.9 (0.7, 1.4)	1.0 (0.8, 1.6)	1.1 (0.8, 1.7)	1.4 (0.9, 2.5)	<0.001
eGFR,mL/min/1.73 m^2^,Median (IQR)	65.5 (37.2, 94.9)	77.0(47.0,98.4)	68.1(43.4,95.7)	66.8(38.9,94.2)	47.8(25.4,90.7)	<0.001
TyG index, Mean ± SD	9.2 ± 0.8	8.2 ± 0.3	8.8 ± 0.1	9.3 ± 0.2	10.3 ± 0.7	<0.001
Hemoglobin,g/dL,Mean ± SD	11.4 ± 2.3	11.4 ± 2.2	11.4 ± 2.4	11.3 ± 2.2	11.3 ± 2.3	0.917
**In the first 2 days after entering ICU**						
Use of high-risk nephrotoxins	841 (59.0)	168 (47.1)	199 (55.9)	221 (62.1)	253 (70.9)	<0.001
Use of vasopressors mediactions	568 (39.8)	113 (31.7)	120 (33.7)	147 (41.3)	188 (52.7)	<0.001
Use of glucocorticoids	288 (20.2)	52 (14.6)	66 (18.5)	84 (23.6)	86 (24.1)	0.004
Use of diuretics	497 (34.9)	118 (33.1)	125 (35.1)	127 (35.7)	127 (35.6)	0.871
Use of acetaminophen	862 (60.4)	230 (64.4)	214 (60.1)	213 (59.8)	205 (57.4)	0.284
Use of albumin 5%/20%	173 (12.1)	42 (11.8)	43 (12.1)	47 (13.2)	41 (11.5)	0.903
Time to extubation ≥24 h	721 (50.6)	168 (47.1)	170 (47.8)	186 (52.2)	197 (55.2)	0.097
**Outcomes**						
SA-AKI^a^,n (%)	1119 (78.5)	254 (71.1)	269 (75.6)	279 (78.4)	317 (88.8)	<0.001
LOS in hopital, day,Median (IQR)	11.1 (6.2, 18.8)	10.0 (5.7, 16.6)	11.8 (6.8, 18.0)	10.8 (6.4, 19.0)	12.6 (6.2, 22.4)	0.019
LOS in ICU, day,Median (IQR)	5.0 (2.7, 9.8)	4.4 (2.2, 7.9)	4.9 (2.8, 8.7)	5.0 (2.6, 10.0)	5.8 (3.0, 12.1)	<0.001

**Abbreviations**: ***CKD***,chronic kidney disease;***CHF,***congestive heart failure;***MI***,myocardial infarction;***SOFA***, sequential organ failure assessment;***CCI***,charlson comorbidity index;***SAPS II***,simplified acute physiology score II;***eGFR***, estimated glomerular filtration rate;***TyG index***,triglyceride-glucose index;***SA-AKI***,sepsis-associated acute kidney injury;***LOS***,length of stay;***ICU***,intensive care unit;***SD***,standard deviation;***IQR***,interquartile range.

a SA-AKI was defined according to KDIGO guidelines as an increase in serum creatinine (Scr) by ≥ 0.3 mg/dl (≥26.5 μmol/l) from baseline within 48h,or urinary output is < 0.5 mL/kg/h for 6 h.

### 3.1 Baseline characteristics

Based on the quartiles of the TyG index, we categorized the patients into four groups with average TyG index values as follows: Q1 8.2 (SD 0.3), Q2 8.8 (SD 0.1), Q3 9.3 (SD 0.2), and Q4 10.3 (SD 0.7). Patient characteristics and outcomes are detailed in Table 1. As the TyG index increased, there was a gradual increase in the proportion of males and those with comorbid diabetes in the study population, along with higher SAPS II and serum creatinine levels. Additionally, individuals with higher TyG index values had a higher proportion of high-risk nephrotoxins, vasopressors medications, and glucocorticoids within the first 48 h of ICU admission (P < 0.01, Table 1).

Table S1 presents the baseline characteristic differences between the AKI and non-AKI groups in this study. Among the patients who developed SA-AKI, the majority were male (56.4%) and white (59.2%), with a higher proportion of comorbidities, such as diabetes, CHF, and MI, compared to the non-AKI group (P < 0.05). Furthermore, patients who developed AKI had longer hospital stays (11.9 days vs. 8.5 days) and ICU stays (5.7 days vs. 2.7 days), a higher average TyG index (9.2 vs. 8.9), and a higher proportion of usage of high-risk nephrotoxins, vasopressors medications, glucocorticoids, diuretics, albumin 5%/20%, and mechanical ventilation for ≥24 h within the first 48 h of ICU admission compared to the non-AKI group (P < 0.05).

Throughout the entire study, the TyG index appeared to be closely associated with patients' comorbidities, disease severity, and laboratory parameters, highlighting its potential value in assessing clinical outcomes.

### 3.2 Primary outcome

In this study, 1119 patients with sepsis developed SA-AKI (78.5%), and the incidence of SA-AKI (71.1% vs. 75.6% vs. 78.4% vs. 88.8%, P < 0.001) gradually increased with an increase in the TyG index (Table 1). Univariate logistic regression analysis was conducted to identify the factors influencing the incidence of AKI in the study population. The results revealed that several variables, including demographics, comorbidities, severity scores, laboratory parameters, medications, and treatments within the first 48 h of ICU admission, were associated with AKI occurrence (Table 2).

**Table 2:** Univariate logistic regression analysis of the factors influencing the incidence of SA-AKI among the study population.

Variables	OR (95%CI)	*P*-value
Sex:female	0.74 (0.57–0.95)	0.019
Age	1.0 (0.99–1.01)	0.804
Insurance:Medicare	1.34 (0.85–2.12)	0.202
Insurance:other	1.27 (0.81–1.98)	0.301
Race:African American	0.88 (0.56–1.40)	0.590
Race:Hispanic or Latino	0.92 (0.43–1.97)	0.831
Race:Asian	0.74 (0.34–1.62)	0.454
Race:Unknown or other	0.98 (0.73–1.31)	0.892
SOFA	1.28 (1.18–1.38)	<0.001
SAPS II	1.05 (1.04–1.06)	<0.001
CCI	1.04 (1.0–1.09)	0.055
Malignant cancer	0.98 (0.64–1.49)	0.926
CKD	1.39 (0.98–1.98)	0.068
Liver disease	1.37 (0.98–1.91)	0.068
Diabetes	1.34 (1.0–1.79)	0.049
Chronic pulmonary disease	1.14 (0.85–1.55)	0.382
MI	1.49 (1.07–2.09)	0.018
CHF	1.98 (1.45–2.70)	<0.001
Hemoglobin	1.01 (0.96–1.07)	0.628
TyG index	1.66 (1.39–1.97)	<0.001
eGFR	0.99 (0.98–0.99)	<0.001
Serum creatinine	1.56 (1.33–1.82)	<0.001
Use of high-risk nephrotoxins	1.36 (1.05–1.75)	0.018
Use of diuretics	1.46 (1.11–1.93)	0.007
Use of glucocorticoids	1.67 (1.18–2.36)	0.004
Use of vasopressors mediactions	3.60 (2.64–4.90)	<0.001
Use of acetaminophen	0.67 (0.51–0.87)	0.003
Use of albumin 5%/20%	3.20 (1.86–5.52)	<0.001
Time to extubation ≥24 h	3.06 (2.33–4.01)	<0.001

**Abbreviations**: ***CKD***,chronic kidney disease;***CHF,***congestive heart failure;***MI***,myocardial infarction;***SOFA***, sequential organ failure assessment;***CCI***,charlson comorbidity index;***SAPS II***,simplified acute physiology score II;***eGFR***, estimated glomerular filtration rate;***TyG index***,triglyceride-glucose index;***SA-AKI***,sepsis-associated acute kidney injury;***OR***,odds ratio;***CI***,confidence interval.

**Fig. 2:**
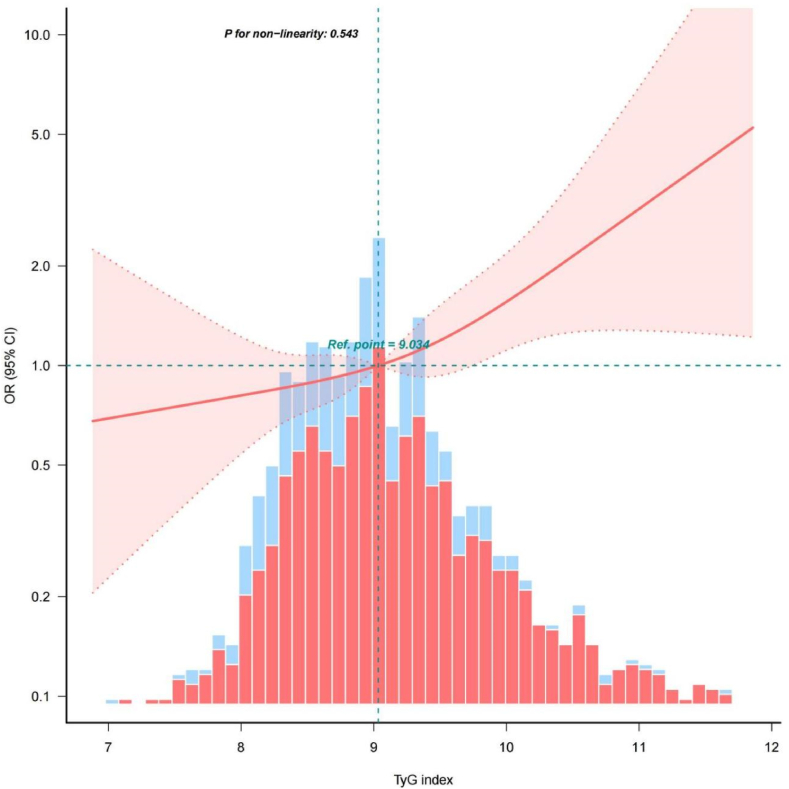
Restricted cubic spline regression analysis of TyG index with SA-AKI.

A multivariable RCS model with full adjustment for clinically relevant covariates, demonstrated a linear relationship between the TyG index and the occurrence of SA-AKI in patients with sepsis (P for non-linearity = 0.543). As the TyG index increased, the incidence of SA-AKI also increased gradually (Fig. 2).

The results of the multivariate logistic regression analysis are presented in Table 3. After adjusting for confounding variables step by step (Models 1, 2, 3, and 4), the TyG index remained independently associated with the incidence of SA-AKI (P < 0.01). When considering all clinical covariates in the adjustment, for each 1-unit increase in the TyG index, the probability of developing SA-AKI within 7 days of ICU admission in critically ill patients increased by 40% (p < 0.001). When the TyG index was used as a categorical variable (categorized by quartiles), with the lowest TyG index group as the reference, the incidence of SA-AKI increased with higher TyG index values in the different groups across various models (P for trend: model 1, P < 0.001; model 2, P < 0.001; model 3, P = 0.004; model 4, P = 0.003).

**Table 3:** Multivariable logistic regression models evaluating the association between TyG index and SA-AKI.

Variable	Events (%)	Model 1		Model 2		Model 3		Model 4	
		OR (95%CI)	*P-value*	OR (95%CI)	*P-value*	OR (95%CI)	*P-value*	OR (95%CI)	*P-value*
TyG index	1119(78.5)	1.66(1.40–1.99)	<0.001	1.63(1.36–1.96)	<0.001	1.37 (1.12–1.68)	0.002	1.40(1.14–1.73)	<0.001
Quartile									
Q1	254 (71.1)	Ref		Ref		Ref		Ref	
Q2	269 (75.6)	1.25(0.90–1.75)	0.190	1.22 (0.87–1.72)	0.249	1.18 (0.82–1.68)	0.373	1.22 (0.84–1.77)	0.290
Q3	279 (78.4)	1.48(1.05–2.09)	0.027	1.37 (0.96–1.96)	0.085	1.25 (0.86–1.83)	0.244	1.28 (0.86–1.90)	0.225
Q4	317 (88.8)	3.26(2.17–4.91)	<0.001	3.18 (2.07–4.87)	<0.001	2.05 (1.3–3.22)	0.002	2.13 (1.33–3.42)	0.002
P for trend			<0.001		<0.001		0.004		0.003

Model 1: adjusted for age,gender, race and insurance.

Model 2: adjusted for Model 1 plus liver disease,CKD,Chronic pulmonary disease,CHF,MI,diabetes and malignant cancer.

Model 3: adjusted for Model 2 plus SOFA, SAPSⅡ,CCI,eGFR, serum creatinine and hemoglobin.

Model 4: adjusted for Model 3 plus use of vasopressors mediactions,use of high-risk nephrotoxins,use of glucocorticoids,use of diuretic,use of acetaminophen,use of albumin 5%/20%,and time to extubation ≥24 h.

**Abbreviations**: ***CKD***,chronic kidney disease;***CHF,***congestive heart failure;***MI***,myocardial infarction;***SOFA***, sequential organ failure assessment;***CCI***,charlson comorbidity index;***SAPS II***,simplified acute physiology score II;***eGFR***, estimated glomerular filtration rate;***TyG index***,triglyceride-glucose index;***SA-AKI***,sepsis-associated acute kidney injury;***OR***,odds ratio;***CI***,confidence interval.

### 3.3 Secondary outcome

In the study population, patients with higher TyG index values had longer hospital stays (12.6 days vs. 10.0 days) and longer ICU stays (5.8 days vs. 4.4 days) (Table 1). Univariate linear regression analysis revealed the effect of various variables on the hospital stay (Tables S2 and S3).

Multiple variable linear regression models were established to assess the relationship between TyG index and LOS, as presented in Table 4. After full adjustment for confounding factors, for each 1-unit increase in the TyG index, the LOS in the hospital increased by 0.79 days, and the LOS in the ICU increased by 0.30 days (P < 0.001). When considering the TyG index as a categorical variable, patients in the high TyG index group had a longer LOS in the hospital and ICU than those in the low TyG index group (P for trend <0.01).

**Table 4:** Multivariable linear regression models evaluating the association between TyG index and LOS.

Variable	Mode 1		Mode 2		Mode 3		Mode 4	
	β (95% CI)	*P-value*	β (95% CI)	*P-value*	β (95% CI)	*P-value*	β (95% CI)	*P-value*
**Length of hospital stay**								
TyG index	1.55(0.65–2.45)	0.001	1.67(0.73–2.61)	0.001	1.68(0.70–2.67)	0.001	1.79(0.80–2.77)	<0.001
Quartile								
Q1	Ref		Ref		Ref		Ref	
Q2	2.66 (0.55–4.76)	0.013	2.86 (0.75–4.98)	0.008	2.81 (0.71–4.92)	0.009	2.96 (0.86–5.06)	0.006
Q3	1.93 (−0.19-4.05)	0.075	2.23 (0.07–4.39)	0.043	2.28 (0.11–4.45)	0.04	2.39 (0.22–4.56)	0.031
Q4	3.32 (1.18–5.45)	0.002	3.58 (1.34–5.83)	0.002	3.29 (0.97–5.61)	0.006	3.51 (1.18–5.83)	0.003
P for trend		0.008		0.005		0.012		0.008
**Length of ICU stay**								
TyG index	1.30(0.84–1.76)	<0.001	1.35(0.87–1.83)	<0.001	1.30(0.80–1.80)	<0.001	1.30(0.80–1.79)	<0.001
Quartile								
Q1	Ref		Ref		Ref		Ref	
Q2	2.66 (0.55–4.76)	0.013	0.68 (−0.4-1.76)	0.216	0.61 (−0.47-1.69)	0.268	0.71 (−0.36-1.77)	0.193
Q3	1.93 (−0.19-4.05)	0.075	1.13 (0.03–2.23)	0.045	1.10 (−0.01-2.21)	0.053	1.13 (0.03–2.23)	0.044
Q4	3.32 (1.18–5.45)	0.002	2.71 (1.56–3.85)	<0.001	2.48 (1.29–3.67)	<0.001	2.43 (1.25–3.61)	<0.001
P for trend		0.008		<0.001		<0.001		<0.001

Model 1: adjusted for age,gender, race and insurance.

Model 2: adjusted for Model 1 plus liver disease,CKD,Chronic pulmonary disease,CHF,MI,diabetes and malignant cancer.

Model 3: adjusted for Model 2 plus SOFA, SAPSⅡ,CCI,eGFR, serum creatinine and hemoglobin.

Model 4: adjusted for Model 3 plus use of vasopressors mediactions,use of high-risk nephrotoxins,use of glucocorticoids,use of diuretic,use of acetaminophen,use of albumin 5%/20%,and time to extubation ≥24 h.

**Abbreviations**: ***CKD***,chronic kidney disease;***CHF,***congestive heart failure;***MI***,myocardial infarction;***SOFA***, sequential organ failure assessment;***CCI***,charlson comorbidity index;***SAPS II***,simplified acute physiology score II;***eGFR***, estimated glomerular filtration rate;***TyG index***,triglyceride-glucose index;***SA-AKI***,sepsis-associated acute kidney injury;***OR***,odds ratio;***CI***,confidence interval.

### 3.4 Subgroup analysis

**Fig. 3:**
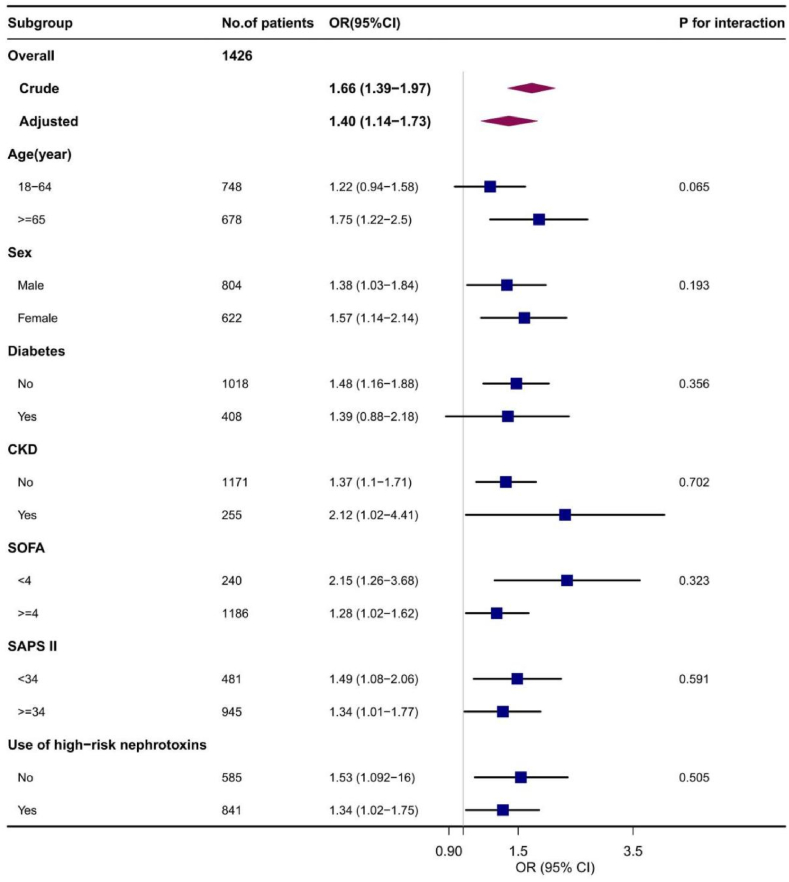
Subgroup and interaction analyses between TyG index and SA-AKI.

**Fig. 4:**
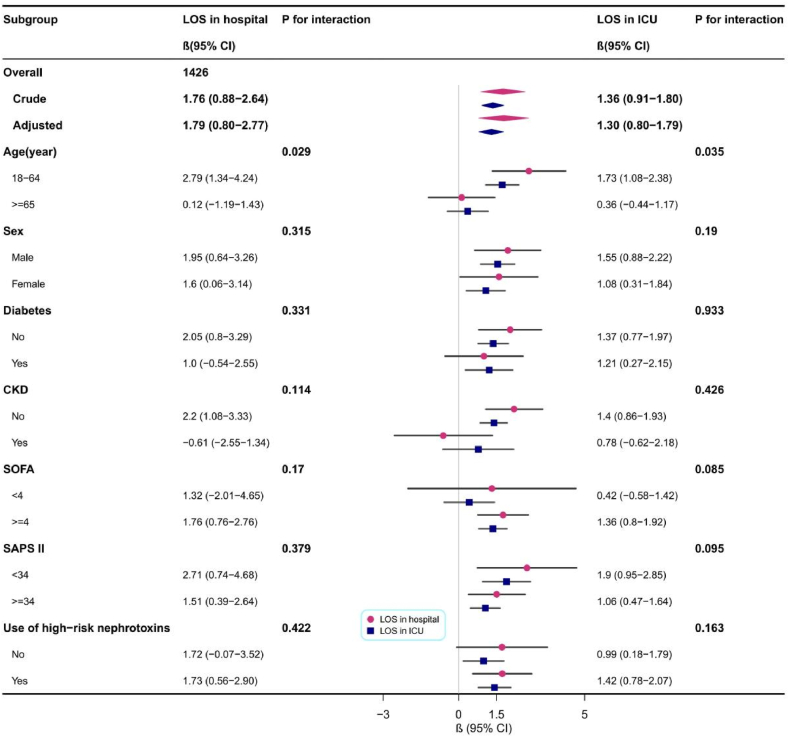
Subgroup and interaction analyses between TyG index and LOS in hospital.

The results of the subgroup analyses are shown in Fig. 3, Fig. 4. These results are consistent with our primary findings. The association between the TyG index and SA-AKI was more pronounced in the subgroups of patients aged ≥65 years (OR 1.75 [95% CI, 1.22–2.5]), those without diabetes (OR 1.48 [95% CI, 1.16–1.88]), and those with SOFA ≤4 (OR 2.15 [95% CI, 1.26–3.68]). No significant interactions were observed in any of the subgroup (P > 0.05).

The correlation between the TyG index and LOS was more significant in the subgroups of patients aged <65 years (LOS in hospital, β, 2.79 [95% CI, 1.34–4.24]; LOS in ICU, β, 1.73 [95% CI, 1.08–2.38]), those without concurrent CKD (LOS in hospital, β, 2.20 [95% CI, 1.08–3.33]; LOS in ICU, β, 1.40 [95% CI, 0.86–1.93]), and those with SOFA ≥4 (LOS in the hospital, β, 1.76 [95% CI, 0.76–2.76]; LOS in the ICU, β, 1.36 [95% CI, 0.80–1.92]). It is worth noting that in adult septic patients aged <65 years, the predictive value of the TyG index for LOS may be more prominent (LOS in the hospital, P for interaction = 0.029; LOS in the ICU, P for interaction = 0.035).

### 3.5 Sensitivity analysis

Table 5 provides the detailed results of the sensitivity analyses. After separately excluding non-White ethnic groups, patients using high-risk nephrotoxins, and patients with concurrent CKD, the association between the TyG index and the incidence of SA-AKI and LOS remained stable. These results are consistent with the primary findings that included all patients.

**Table 5:** Sensitivity analyses.

Analysis	Events	Unadjusted model		Adjusted model^a^	
		OR (95%CI)	*P-value*	OR (95%CI)	*P-value*
**Any-stage SA-AKI**					
**Excluding participants using high-risk nephrotoxins**					
TyG index	585	1.73 (1.29–2.31)	<0.001	1.57 (1.13–2.2)	0.008
**Excluding non-White participants**					
TyG index	844	1.65 (1.31–2.06)	<0.001	1.44 (1.1–1.87)	0.007
**Excluding participants with CKD**					
TyG index	1171	1.61 (1.34–1.94)	<0.001	1.36 (1.09–1.69)	0.006
**Length of hospital stay**^b^					
**Excluding participants using high-risk nephrotoxins**					
TyG index	585	1.38 (−0.25-3.01)	0.098	1.83 (0.03–3.64)	0.047
**Excluding non-White participants**					
TyG index	844	1.85 (0.81–2.9)	0.001	1.51 (0.35–2.66)	0.011
**Excluding participants with CKD**					
TyG index	1171	1.97 (0.96–2.99)	<0.001	2.09 (0.97–3.22)	<0.001
**Length of ICU stay**^b^					
**Excluding participants using high-risk nephrotoxins**					
TyG index	585	0.87 (0.14–1.61)	0.021	1 (0.21–1.8)	0.013
**Excluding non-White participants**					
TyG index	844	1.39 (0.86–1.92)	<0.001	1.23 (0.64–1.81)	<0.001
**Excluding participants with CKD**					
TyG index	1171	1.41 (0.92–1.89)	<0.001	1.37 (0.83–1.9)	<0.001

**Abbreviations**: ***CHF,***congestive heart failure;***MI***,myocardial infarction;***SOFA***, sequential organ failure assessment;***CCI***,charlson comorbidity index;***SAPS II***,simplified acute physiology score II;***eGFR***, estimated glomerular filtration rate;***TyG index***,triglyceride-glucose index;***SA-AKI***,sepsis-associated acute kidney injury;***OR***,odds ratio;***CI***,confidence interval.

a Adjusted for age,sex,insurance, liver disease, chronic pulmonary disease,CHF,MI,diabetes, malignant cancer, SOFA,hemoglobin, serum creatinine, eGFR,CCI,SAPSⅡ,use of vasopressors mediactions,use of glucocorticoids,use of diuretic,use of acetaminophen,use of albumin 5%/20%,and time to extubation ≥24 h.

b Linear regression was used to evaluate the association between TyG index and length of stay. The results were expressed as β (95% CIs).

## 4 Discussion

This study represents the first exploration of the predictive value of TyG index for SA-AKI and LOS in patients with sepsis. After adjusting for potential confounders, TyG index exhibited a linear relationship with SA-AKI. It remained independently associated with SA-AKI and LOS as both continuous and categorical variables when the quartiles were considered. These associations were consistent across the subgroup and sensitivity analyses. These findings suggest that for septic patients with significantly elevated TyG index values, early intervention may reduce the incidence of SA-AKI or delay its progression, leading to a shorter hospital stay.

### 4.1 Sepsis and SA-AKI

This study extracted pertinent data on the occurrence of AKI in sepsis patients within seven days of ICU admission. Globally, there are approximately 19 million cases of sepsis each year, and it is noted that roughly two-thirds of septic shock patients develop AKI [8,9,31]. The epidemiology of SA-AKI exhibits significant variations and lacks standardized definitions [32]. The latest consensus defines SA-AKI as the occurrence of AKI within seven days of sepsis diagnosis. The diagnostic criteria for sepsis and AKI were defined according to the recommendations provided by Sepsis-3 and Kidney Disease: Improving Global Outcomes [29].SA-AKI exhibits a high degree of heterogeneity owing to various mechanisms in patients with sepsis, including direct infection, infection-related responses, and adverse effects of treatment, resulting in varying degrees of injury [33]. The reported mechanisms of SA-AKI injury primarily include systemic and renal inflammatory responses, mitochondrial dysfunction, complement activation, renin-angiotensin-aldosterone system(RAAS) dysregulation, metabolic reprogramming, microcirculatory dysfunction, and hemodynamic abnormalities [29,[34], [35], [36]].

Previous studies have suggested that risk factors for SA-AKI include the use of vasopressors, nephrotoxic medications, mechanical ventilation, the presence of liver disease, congestive heart failure, malignant tumors, CKD, and diabetes [29,[37], [38], [39]].Recent research indicates that early use of acetaminophen after cardiac surgery can reduce the risk of severe AKI in patients [40].However, research on SA-AKI interventions has suggested that the use of anti-inflammatory drugs does not have a significant advantage in preventing or treating SA-AKI. However, the use of dexamethasone may reduce the need for renal replacement therapy in patients [41]. To minimize bias, we extracted data on the use of vasopressors, nephrotoxic medications, glucocorticoids, and mechanical ventilation for ≥24 h within the first two days of ICU admission and adjusted for these factors along with the presence of liver disease, CKD, CHF, malignant cancer, and diabetes as potential confounders based on the aforementioned research results.

The diagnosis of SA-AKI is increasingly emphasizing the early assessment of renal function and definitive diagnosis through the use of biomarkers, in addition to established criteria such as KDIGO and Sepsis 3.0, to improve patient outcomes [42]. The utility of α1-microglobulin and urine microscopy score as indicators for the early prediction of AKI is yet to be validated [43,44]. The elevation of the AKI damage marker neutrophil gelatinase-associated lipocalin (NGAL) in sepsis and inflammation restricts its clinical utility in predicting SA-AKI [45,46].Other AKI markers, such as intrarenal venous flow and kidney injury molecule 1, suffer from limitations including significant observation bias and low sensitivity, making them less suitable for widespread clinical application [47,48]. Therefore, it is imperative to identify a new marker for the early prediction of SA-AKI risk in patients with sepsis.

The cytokine storm associated with sepsis triggers immune-mediated inflammatory dyslipidemia [19]. Recent research has identified a correlation between the TyG index and AKI in critically ill patients in the ICU [49]. However, whether TyG index can predict SA-AKI in specific sepsis populations requires further validation. In our initial hypothesis, we assumed that the TyG index could serve as an early predictor of SA-AKI and impact the LOS.

### 4.2 TyG index and possible mechanisms

The TyG index has been validated as a simple surrogate marker for IR, a key mediator in the pathogenesis of type 2 diabetes [50,51]. In addition to diabetes, the TyG index has also been reported to be associated with the occurrence or development of metabolic syndrome, cardiovascular disease, metabolic dysfunction-associated fatty liver disease, ischemic stroke, chronic kidney disease, cerebrovascular disease, subclinical atherosclerosis, and arterial stiffness [[50], [51], [52], [53], [54], [55], [56], [57], [58], [59], [60]]. Furthermore, in critically ill patients, the TyG index has been associated with all-cause mortality in patients with concomitant ischemic stroke, chronic kidney disease, and coronary artery disease [[61], [62], [63], [64]]. Considering that the TyG index may be related to chronic kidney disease (CKD), which is a potential risk factor for SA-AKI, we conducted a subgroup analysis among patients with concomitant CKD and performed a sensitivity analysis by excluding those with CKD. The results showed that the association between TyG index and SA-AKI remained consistent with the main findings (CKD subgroup analysis, OR 2.12 [95% CI, 1.02–4.41], P for interaction = 0.702; sensitivity analysis, OR 1.36 [95% CI, 1.09–1.69]). These findings highlight the predictive value of the TyG index in critically ill patients, and suggest that its association with other diseases or pathological conditions warrants further research.

A series of studies on the impact of TyG index on renal function that have been reported can contribute to further exploration of its potential mechanisms in SA-AKI. First, oxidative stress induced by insulin resistance is closely associated with endothelial cell damage in renal glomeruli, thickening of the basement membrane, and proliferation of mesangial cells, which can lead to glomerulosclerosis and damage to the renal tubulointerstitium, ultimately resulting in renal dysfunction [65]. Second, in a state of hyperinsulinemia induced by insulin resistance, insulin can promote sodium reabsorption and increase glomerular filtration rate, which can lead to renal damage [66,67]. Finally, insulin resistance may lead to increased catecholamine levels and dysregulation of pro-inflammatory and adipokines, which could lead to chronic hypercatecholaminemia and inflammation, thus adversely affecting renal function [68,69]. High TyG index may be associated with the development of SA-AKI and progression of ischemic stroke in patients, thereby prolonging hospitalization and ICU stay.

### 4.3 Study limitations

First, this study was a single-center investigation; however, the substantial sample size and comprehensive data from the MIMIC-IV database contributed to its representativeness. While the majority of the study population consisted of Caucasians, we conducted a separate sensitivity analysis within the white population, which yielded results consistent with the main findings.Second, there was a notable amount of missing triglyceride data for ICU-admitted patients in the MIMIC-IV database. Nevertheless, due to the substantial sample size, we were able to include complete TyG index data for 1426 septic patients in our analysis. Third, we did not validate the association of other metrics evaluating individual IR levels (e.g., Homeostasis Model Assessment-Insulin Resistance index) with the development of SA-AKI, which may need to be further investigated by designing prospective clinical trials. Finally, this study is retrospective in nature, and there may be unadjusted confounding factors that could influence the outcomes. We conducted subgroup analyses and a series of sensitivity analyses to examine the stability and consistency of the results from different perspectives. It is noteworthy that in this study, we adjusted for the use of various medications, including high-risk nephrotoxins, diuretics, glucocorticoids, and vasopressors medications, among others (specific medication types are detailed in Table S5). This is different from many previous related studies that did not account for the use of medications that could potentially impact AKI or prognosis in critically ill patients, which might affect the stability of the results [28,[61], [62], [63], [64],^69^,^70^].

## 5 Conclusions

In this study, we have established a significant association between the TyG index and SA-AKI as well as LOS, suggesting that the TyG index could serve as a potential predictive marker for SA-AKI and LOS in septic patients. Our findings expand the utility of the TyG index in patients with sepsis and monitoring the TyG index may contribute to clinical decision-making and disease management. Further research is needed to determine whether interventions targeting the TyG index can improve the clinical outcomes in patients with sepsis.

## Data availability

The datasets generated and analyzed during the current study can be obtained from the corresponding author upon reasonable request.

## Funding

NA.

## Ethical considerations and informed consent

This study adhered to the principles outlined in the Declaration of Helsinki. Furthermore, it is important to note that the MIMIC-IV database received approval from the Massachusetts Institute of Technology and the Beth Israel Deaconess Medical Center. The copies of the datasets used in this study are available from the MIMIC database. Consequently, this research trial did not require ethical approval or the acquisition of informed consent.

## Consent for publication

Not applicable.

## CRediT authorship contribution statement

**Yijiao Fang:** Writing – original draft, Methodology, Data curation. **Bo Xiong:** Visualization, Software, Investigation. **Xue Shang:** Visualization, Software, Investigation. **Fan Yang:** Validation, Software, Methodology. **Yuehao Yin:** Formal analysis. **Zhirong Sun:** Supervision. **Xin Wu:** Supervision, Formal analysis. **Jun Zhang:** Writing – review & editing, Supervision, Methodology, Conceptualization. **Yi Liu:** Writing – review & editing, Validation, Resources, Project administration, Conceptualization.

## Declaration of competing interest

The authors declare that they have no known competing financial interests or personal relationships that could have appeared to influence the work reported in this paper.
