# Supplementary material for: Triglyceride-glucose index predicts sepsis-associated acute kidney injury and length of stay in sepsis: A MIMIC-IV cohort study
Source: Heliyon. 2024 Apr 4;10(7):e29257. doi: 10.1016/j.heliyon.2024.e29257 (PMC11015450; doi:10.1016/j.heliyon.2024.e29257)
Supplement: Multimedia component 1 [file mmc1.docx]

**Tables**

**Table 1.** Baseline characteristics and outcomes of sepsis patients grouped according to TyG index quartiles.

| **Categories** | **Overall(N=1426)** | **Q1 (N = 357)** | **Q2 (n = 356)** | **Q3 (n = 356)** | **Q4 (n = 357)** | ***P*-value** |
| --- | --- | --- | --- | --- | --- | --- |
| **Demographic** |  |  |  |  |  |  |
| Age,year,Mean±SD | 62.1 ± 17.5 | 65.8 ± 18.9 | 64.6 ± 16.6 | 61.1 ± 16.9 | 57.0 ± 16.0 | < 0.001 |
| Sex, n (%) |  |  |  |  |  | 0.084 |
| Male | 804 (56.4) | 183 (51.3) | 198 (55.6) | 208 (58.4) | 215 (60.2) |  |
| Female | 622 (43.6) | 174 (48.7) | 158 (44.4) | 148 (41.6) | 142 (39.8) |  |
| Insurance, n (%) |  |  |  |  |  | 0.028 |
| Medicaid | 120 ( 8.4) | 28 (7.8) | 27 (7.6) | 35 (9.8) | 30 (8.4) |  |
| Medicare | 588 (41.2) | 165 (46.2) | 150 (42.1) | 152 (42.7) | 121 (33.9) |  |
| Other | 718 (50.4) | 164 (45.9) | 179 (50.3) | 169 (47.5) | 206 (57.7) |  |
| Race, n (%) |  |  |  |  |  | 0.225 |
| White | 844 (59.2) | 216 (60.5) | 216 (60.7) | 202 (56.7) | 210 (58.8) |  |
| African American | 116 ( 8.1) | 36 (10.1) | 29 (8.1) | 27 (7.6) | 24 (6.7) |  |
| Hispanic or Latino | 40 ( 2.8) | 7 (2) | 9 (2.5) | 17 (4.8) | 7 (2) |  |
| Asian | 34 ( 2.4) | 6 (1.7) | 8 (2.2) | 13 (3.7) | 7 (2) |  |
| Unknown or other | 392 (27.5) | 92 (25.8) | 94 (26.4) | 97 (27.2) | 109 (30.5) |  |
| **Comorbidities** |  |  |  |  |  |  |
| CKD,n (%) | 255 (17.9) | 51 (14.3) | 77 (21.6) | 61 (17.1) | 66 (18.5) | 0.080 |
| Chronic pulmonary disease,n (%) | 338 (23.7) | 78 (21.8) | 87 (24.4) | 86 (24.2) | 87 (24.4) | 0.822 |
| Liver disease,n (%) | 285 (20.0) | 64 (17.9) | 56 (15.7) | 85 (23.9) | 80 (22.4) | 0.022 |
| Diabetes,n (%) | 408 (28.6) | 44 (12.3) | 82 (23) | 117 (32.9) | 165 (46.2) | < 0.001 |
| Malignant cancer,n (%) | 142 (10.0) | 32 (9) | 34 (9.6) | 37 (10.4) | 39 (10.9) | 0.824 |
| CHF,n (%) | 411 (28.8) | 95 (26.6) | 105 (29.5) | 116 (32.6) | 95 (26.6) | 0.237 |
| MI,n (%) | 302 (21.2) | 61 (17.1) | 78 (21.9) | 90 (25.3) | 73 (20.4) | 0.060 |
| **Severity scores** |  |  |  |  |  |  |
| SOFA, Median (IQR) | 3.0 (2.0, 4.0) | 3.0 (2.0, 4.0) | 3.0 (2.0, 4.0) | 3.0 (2.0, 5.0) | 4.0 (2.0, 6.0) | < 0.001 |
| CCI, Median (IQR) | 6.0 (4.0, 8.0) | 6.0 (4.0, 8.0) | 6.0 (4.0, 8.0) | 6.0 (4.0, 8.0) | 5.0 (3.0, 7.0) | 0.012 |
| SAPS II, Mean ± SD | 40.8 ± 15.1 | 38.8 ± 13.2 | 39.2 ± 14.4 | 39.9 ± 14.9 | 45.2 ± 16.8 | < 0.001 |
| **Laboratory parameters** |  |  |  |  |  |  |
| Serum creatinine,mg/dL,Median (IQR) | 1.1 (0.8, 1.8) | 0.9 (0.7, 1.4) | 1.0 (0.8, 1.6) | 1.1 (0.8, 1.7) | 1.4 (0.9, 2.5) | < 0.001 |
| eGFR,mL/min/1.73 m^2^,Median (IQR) | 65.5 (37.2, 94.9) | 77.0(47.0,98.4) | 68.1(43.4,95.7) | 66.8(38.9,94.2) | 47.8(25.4,90.7) | < 0.001 |
| TyG index, Mean ± SD | 9.2 ± 0.8 | 8.2 ± 0.3 | 8.8 ± 0.1 | 9.3 ± 0.2 | 10.3 ± 0.7 | < 0.001 |
| Hemoglobin,g/dL,Mean ± SD | 11.4 ± 2.3 | 11.4 ± 2.2 | 11.4 ± 2.4 | 11.3 ± 2.2 | 11.3 ± 2.3 | 0.917 |
| **In the first 2 days after entering ICU** |  |  |  |  |  |  |
| Use of high-risk nephrotoxins | 841 (59.0) | 168 (47.1) | 199 (55.9) | 221 (62.1) | 253 (70.9) | < 0.001 |
| Use of vasopressors mediactions | 568 (39.8) | 113 (31.7) | 120 (33.7) | 147 (41.3) | 188 (52.7) | < 0.001 |
| Use of glucocorticoids | 288 (20.2) | 52 (14.6) | 66 (18.5) | 84 (23.6) | 86 (24.1) | 0.004 |
| Use of diuretics | 497 (34.9) | 118 (33.1) | 125 (35.1) | 127 (35.7) | 127 (35.6) | 0.871 |
| Use of acetaminophen | 862 (60.4) | 230 (64.4) | 214 (60.1) | 213 (59.8) | 205 (57.4) | 0.284 |
| Use of albumin 5%/20% | 173 (12.1) | 42 (11.8) | 43 (12.1) | 47 (13.2) | 41 (11.5) | 0.903 |
| Time to extubation ≥24 h | 721 (50.6) | 168 (47.1) | 170 (47.8) | 186 (52.2) | 197 (55.2) | 0.097 |
| **Outcomes** |  |  |  |  |  |  |
| SA-AKI^a^ ,n (%) | 1119 (78.5) | 254 (71.1) | 269 (75.6) | 279 (78.4) | 317 (88.8) | < 0.001 |
| LOS in hopital, day,Median (IQR) | 11.1 (6.2, 18.8) | 10.0 (5.7, 16.6) | 11.8 (6.8, 18.0) | 10.8 (6.4, 19.0) | 12.6 (6.2, 22.4) | 0.019 |
| LOS in ICU, day,Median (IQR) | 5.0 (2.7, 9.8) | 4.4 (2.2, 7.9) | 4.9 (2.8, 8.7) | 5.0 (2.6, 10.0) | 5.8 (3.0, 12.1) | < 0.001 |

**Abbreviations**: ***CKD***,chronic kidney disease;***CHF,***congestive heart failure;***MI***,myocardial infarction;***SOFA***,sequential organ failure assessment;***CCI***,charlson comorbidity index;***SAPS II***,simplified acute physiology score II;***eGFR***,estimated glomerular filtration rate;***TyG index***,triglyceride-glucose index;***SA-AKI***,sepsis-associated acute kidney injury;***LOS***,length of stay;***ICU***,intensive care unit;***SD***,standard deviation;***IQR***,interquartile range.

^a^ SA-AKI was defined according to KDIGO guidelines as an increase in serum creatinine (Scr) by ≥0.3 mg/dl (≥26.5μmol/l) from baseline within 48h,or urinary output is <0.5 mL/kg/h for 6 h.

**Table 2.** Univariate logistic regression analysis of the factors influencing the incidence of SA-AKI among the study population.

| **Variables** | **OR (95%CI)** | ***P*-value** |
| --- | --- | --- |
| Sex:female | 0.74 (0.57-0.95) | 0.019 |
| Age | 1.0 (0.99-1.01) | 0.804 |
| Insurance:Medicare | 1.34 (0.85-2.12) | 0.202 |
| Insurance:other | 1.27 (0.81-1.98) | 0.301 |
| Race:African American | 0.88 (0.56-1.40) | 0.590 |
| Race:Hispanic or Latino | 0.92 (0.43-1.97) | 0.831 |
| Race:Asian | 0.74 (0.34-1.62) | 0.454 |
| Race:Unknown or other | 0.98 (0.73-1.31) | 0.892 |
| SOFA | 1.28 (1.18-1.38) | <0.001 |
| SAPS II | 1.05 (1.04-1.06) | <0.001 |
| CCI | 1.04 (1.0-1.09) | 0.055 |
| Malignant cancer | 0.98 (0.64-1.49) | 0.926 |
| CKD | 1.39 (0.98-1.98) | 0.068 |
| Liver disease | 1.37 (0.98-1.91) | 0.068 |
| Diabetes | 1.34 (1.0-1.79) | 0.049 |
| Chronic pulmonary disease | 1.14 (0.85-1.55) | 0.382 |
| MI | 1.49 (1.07-2.09) | 0.018 |
| CHF | 1.98 (1.45-2.70) | <0.001 |
| Hemoglobin | 1.01 (0.96-1.07) | 0.628 |
| TyG index | 1.66 (1.39-1.97) | <0.001 |
| eGFR | 0.99 (0.98-0.99) | <0.001 |
| Serum creatinine | 1.56 (1.33-1.82) | <0.001 |
| Use of high-risk nephrotoxins | 1.36 (1.05-1.75) | 0.018 |
| Use of diuretics | 1.46 (1.11-1.93) | 0.007 |
| Use of glucocorticoids | 1.67 (1.18-2.36) | 0.004 |
| Use of vasopressors mediactions | 3.60 (2.64-4.90) | <0.001 |
| Use of acetaminophen | 0.67 (0.51-0.87) | 0.003 |
| Use of albumin 5%/20% | 3.20 (1.86-5.52) | <0.001 |
| Time to extubation ≥24 h | 3.06 (2.33-4.01) | <0.001 |

**Abbreviations**: ***CKD***,chronic kidney disease;***CHF,***congestive heart failure;***MI***,myocardial infarction;***SOFA***,sequential organ failure assessment;***CCI***,charlson comorbidity index;***SAPS II***,simplified acute physiology score II;***eGFR***,estimated glomerular filtration rate;***TyG index***,triglyceride-glucose index;***SA-AKI***,sepsis-associated acute kidney injury;***OR***,odds ratio;***CI***,confidence interval.

**Table 3.** Multivariable logistic regression models evaluating the association between TyG index and SA-AKI.

| **Variable** | **Events**  **(%)** | **Model 1** | | **Model 2** | | **Model 3** | | **Model 4** | |
| --- | --- | --- | --- | --- | --- | --- | --- | --- | --- |
|  |  | **OR (95%CI)** | ***P-value*** | **OR (95%CI)** | ***P-value*** | **OR (95%CI)** | ***P-value*** | **OR (95%CI)** | ***P-value*** |
| TyG index | 1119(78.5) | 1.66(1.40-1.99) | <0.001 | 1.63(1.36-1.96) | <0.001 | 1.37 (1.12-1.68) | 0.002 | 1.40(1.14-1.73) | <0.001 |
| Quartile |  |  |  |  |  |  |  |  |  |
| Q1 | 254 (71.1) | Ref |  | Ref |  | Ref |  | Ref |  |
| Q2 | 269 (75.6) | 1.25(0.90-1.75) | 0.190 | 1.22 (0.87-1.72) | 0.249 | 1.18 (0.82-1.68) | 0.373 | 1.22 (0.84-1.77) | 0.290 |
| Q3 | 279 (78.4) | 1.48(1.05-2.09) | 0.027 | 1.37 (0.96-1.96) | 0.085 | 1.25 (0.86-1.83) | 0.244 | 1.28 (0.86-1.90) | 0.225 |
| Q4 | 317 (88.8) | 3.26(2.17-4.91) | <0.001 | 3.18 (2.07-4.87) | <0.001 | 2.05 (1.3-3.22) | 0.002 | 2.13 (1.33-3.42) | 0.002 |
| P for trend |  |  | <0.001 |  | <0.001 |  | 0.004 |  | 0.003 |

Model 1: adjusted for age,gender,race and insurance.

Model 2: adjusted for Model 1 plus liver disease,CKD,Chronic pulmonary disease,CHF,MI,diabetes and malignant cancer.

Model 3: adjusted for Model 2 plus SOFA,SAPSⅡ,CCI,eGFR,serum creatinine and hemoglobin.

Model 4: adjusted for Model 3 plus use of vasopressors mediactions,use of high-risk nephrotoxins,use of glucocorticoids,use of diuretic,use of acetaminophen,use of albumin 5%/20%,and time to extubation ≥24 h.

**Abbreviations**: ***CKD***,chronic kidney disease;***CHF,***congestive heart failure;***MI***,myocardial infarction;***SOFA***,sequential organ failure assessment;***CCI***,charlson comorbidity index;***SAPS II***,simplified acute physiology score II;***eGFR***,estimated glomerular filtration rate;***TyG index***,triglyceride-glucose index;***SA-AKI***,sepsis-associated acute kidney injury;***OR***,odds ratio;***CI***,confidence interval.

**Table 4.** Multivariable linear regression models evaluating the association between TyG index and LOS.

| **Variable** |  | **Mode 1** | | **Mode 2** | | **Mode 3** | | **Mode 4** | |
| --- | --- | --- | --- | --- | --- | --- | --- | --- | --- |
|  |  | **β (95% CI)** | ***P-value*** | **β (95% CI)** | ***P-value*** | **β (95% CI)** | ***P-value*** | **β (95% CI)** | ***P-value*** |
| **Length of hospital stay** | | |  |  |  |  |  |  |  |
| TyG index |  | 1.55(0.65-2.45) | 0.001 | 1.67(0.73-2.61) | 0.001 | 1.68(0.70-2.67) | 0.001 | 1.79(0.80-2.77) | <0.001 |
| Quartile |  |  |  |  |  |  |  |  |  |
| Q1 |  | Ref |  | Ref |  | Ref |  | Ref |  |
| Q2 |  | 2.66 (0.55-4.76) | 0.013 | 2.86 (0.75-4.98) | 0.008 | 2.81 (0.71-4.92) | 0.009 | 2.96 (0.86-5.06) | 0.006 |
| Q3 |  | 1.93 (-0.19-4.05) | 0.075 | 2.23 (0.07-4.39) | 0.043 | 2.28 (0.11-4.45) | 0.04 | 2.39 (0.22-4.56) | 0.031 |
| Q4 |  | 3.32 (1.18-5.45) | 0.002 | 3.58 (1.34-5.83) | 0.002 | 3.29 (0.97-5.61) | 0.006 | 3.51 (1.18-5.83) | 0.003 |
| P for trend |  |  | 0.008 |  | 0.005 |  | 0.012 |  | 0.008 |
| **Length of ICU stay** | | |  |  |  |  |  |  |  |
| TyG index |  | 1.30(0.84-1.76) | <0.001 | 1.35(0.87-1.83) | <0.001 | 1.30(0.80-1.80) | <0.001 | 1.30(0.80-1.79) | <0.001 |
| Quartile |  |  |  |  |  |  |  |  |  |
| Q1 |  | Ref |  | Ref |  | Ref |  | Ref |  |
| Q2 |  | 2.66 (0.55-4.76) | 0.013 | 0.68 (-0.4-1.76) | 0.216 | 0.61 (-0.47-1.69) | 0.268 | 0.71 (-0.36-1.77) | 0.193 |
| Q3 |  | 1.93 (-0.19-4.05) | 0.075 | 1.13 (0.03-2.23) | 0.045 | 1.10 (-0.01-2.21) | 0.053 | 1.13 (0.03-2.23) | 0.044 |
| Q4 |  | 3.32 (1.18-5.45) | 0.002 | 2.71 (1.56-3.85) | <0.001 | 2.48 (1.29-3.67) | <0.001 | 2.43 (1.25-3.61) | <0.001 |
| P for trend |  |  | 0.008 |  | <0.001 |  | <0.001 |  | <0.001 |

Model 1: adjusted for age,gender,race and insurance.

Model 2: adjusted for Model 1 plus liver disease,CKD,Chronic pulmonary disease,CHF,MI,diabetes and malignant cancer.

Model 3: adjusted for Model 2 plus SOFA,SAPSⅡ,CCI,eGFR,serum creatinine and hemoglobin.

Model 4: adjusted for Model 3 plus use of vasopressors mediactions,use of high-risk nephrotoxins,use of glucocorticoids,use of diuretic,use of acetaminophen,use of albumin 5%/20%,and time to extubation ≥24 h.

**Abbreviations**: ***CKD***,chronic kidney disease;***CHF,***congestive heart failure;***MI***,myocardial infarction;***SOFA***,sequential organ failure assessment;***CCI***,charlson comorbidity index;***SAPS II***,simplified acute physiology score II;***eGFR***,estimated glomerular filtration rate;***TyG index***,triglyceride-glucose index;***SA-AKI***,sepsis-associated acute kidney injury;***OR***,odds ratio;***CI***,confidence interval.

**Table 5.** Sensitivity Analyses.

| **Analysis** | **Events** | **Unadjusted model** | |  | **Adjusted model**a | |
| --- | --- | --- | --- | --- | --- | --- |
|  |  | **OR (95%CI)** | ***P-value*** |  | **OR (95%CI)** | ***P-value*** |
| **Any-stage SA-AKI** |  |  |  |  |  |  |
| **Excluding participants using high-risk nephrotoxins** | | | |  |  |  |
| TyG index | 585 | 1.73 (1.29-2.31) | <0.001 |  | 1.57 (1.13-2.2) | 0.008 |
| **Excluding non-White participants** | | |  |  |  |  |
| TyG index | 844 | 1.65 (1.31-2.06) | <0.001 |  | 1.44 (1.1-1.87) | 0.007 |
| **Excluding participants with CKD** | | | |  |  |  |
| TyG index | 1171 | 1.61 (1.34-1.94) | <0.001 |  | 1.36 (1.09-1.69) | 0.006 |
| **Length of hospital stay^b^** | | | |  |  |  |
| **Excluding participants using high-risk nephrotoxins** | | | |  |  |  |
| TyG index | 585 | 1.38 (-0.25-3.01) | 0.098 |  | 1.83 (0.03-3.64) | 0.047 |
| **Excluding non-White participants** | | | |  |  |  |
| TyG index | 844 | 1.85 (0.81-2.9) | 0.001 |  | 1.51 (0.35-2.66) | 0.011 |
| **Excluding participants with CKD** | | | |  |  |  |
| TyG index | 1171 | 1.97 (0.96-2.99) | <0.001 |  | 2.09 (0.97-3.22) | <0.001 |
| **Length of ICU stay**b |  |  |  |  |  |  |
| **Excluding participants using high-risk nephrotoxins** | | | |  |  |  |
| TyG index | 585 | 0.87 (0.14-1.61) | 0.021 |  | 1 (0.21-1.8) | 0.013 |
| **Excluding non-White participants** | | | |  |  |  |
| TyG index | 844 | 1.39 (0.86-1.92) | <0.001 |  | 1.23 (0.64-1.81) | <0.001 |
| **Excluding participants with CKD** | | | |  |  |  |
| TyG index | 1171 | 1.41 (0.92-1.89) | <0.001 |  | 1.37 (0.83-1.9) | <0.001 |

**^a^** Adjusted for age,sex,insurance,liver disease,chronic pulmonary disease,CHF,MI,diabetes,malignant cancer,SOFA,hemoglobin,serum creatinine,eGFR,CCI,SAPSⅡ,use of vasopressors mediactions,use of glucocorticoids,use of diuretic,use of acetaminophen,use of albumin 5%/20%,and time to extubation ≥24 h.

**^b^** Linear regression was used to evaluate the association between TyG index and length of stay. The results were expressed as β (95% CIs).

**Abbreviations**: ***CHF,***congestive heart failure;***MI***,myocardial infarction;***SOFA***,sequential organ failure assessment;***CCI***,charlson comorbidity index;***SAPS II***,simplified acute physiology score II;***eGFR***,estimated glomerular filtration rate;***TyG index***,triglyceride-glucose index;***SA-AKI***,sepsis-associated acute kidney injury;***OR***,odds ratio;***CI***,confidence interval.

**Table S1.** Baseline characteristics and outcomes of sepsis patients grouped according to SA-AKI^a^ .

| **Categories** | **Overall (N=1426)** | **Non-SA-AKI (n=307)** | **SA-AKI (n=1119)** | ***P*-value** |
| --- | --- | --- | --- | --- |
| **Demographic** |  |  |  |  |
| Age,year,Mean±SD | 62.1 ± 17.5 | 62.3 ± 19.5 | 62.0 ± 16.8 | 0.805 |
| Sex, n (%) |  |  |  | 0.019 |
| Male | 804 (56.4) | 155 (50.5) | 649 (58) |  |
| Female | 622 (43.6) | 152 (49.5) | 470 (42) |  |
| Insurance, n (%) |  |  |  | 0.442 |
| Medicaid | 120 ( 8.4) | 31 (10.1) | 89 (8) |  |
| Medicare | 588 (41.2) | 121 (39.4) | 467 (41.7) |  |
| Other | 718 (50.4) | 155 (50.5) | 563 (50.3) |  |
| Race, n (%) |  |  |  | 0.935 |
| White | 844 (59.2) | 178 (58) | 666 (59.5) |  |
| African American | 116 ( 8.1) | 27 (8.8) | 89 (8) |  |
| Hispanic or Latino | 40 ( 2.8) | 9 (2.9) | 31 (2.8) |  |
| Asian | 34 ( 2.4) | 9 (2.9) | 25 (2.2) |  |
| Unknown or other | 392 (27.5) | 84 (27.4) | 308 (27.5) |  |
| **Comorbidities** |  |  |  |  |
| CKD,n (%) | 255 (17.9) | 44 (14.3) | 211 (18.9) | 0.067 |
| Chronic pulmonary disease,n (%) | 338 (23.7) | 67 (21.8) | 271 (24.2) | 0.382 |
| Liver disease,n (%) | 285 (20.0) | 50 (16.3) | 235 (21) | 0.067 |
| Diabetes,n (%) | 408 (28.6) | 74 (24.1) | 334 (29.8) | 0.049 |
| Malignant cancer,n (%) | 142 (10.0) | 31 (10.1) | 111 (9.9) | 0.926 |
| CHF,n (%) | 411 (28.8) | 58 (18.9) | 353 (31.5) | < 0.001 |
| MI,n (%) | 302 (21.2) | 50 (16.3) | 252 (22.5) | 0.018 |
| **Severity scores** |  |  |  |  |
| SOFA, Median (IQR) | 3.0 (2.0, 4.0) | 2.0 (2.0, 4.0) | 3.0 (2.0, 5.0) | < 0.001 |
| CCI, Median (IQR) | 6.0 (4.0, 8.0) | 6.0 (3.0, 8.0) | 6.0 (4.0, 8.0) | 0.157 |
| SAPS II, Mean ± SD | 40.8 ± 15.1 | 33.4 ± 11.4 | 42.8 ± 15.4 | < 0.001 |
| **Laboratory parameters** |  |  |  |  |
| Serum creatinine,mg/dL,Median (IQR) | 1.1 (0.8, 1.8) | 0.9 (0.7, 1.3) | 1.1 (0.8, 2.0) | < 0.001 |
| eGFR, Median (IQR) | 65.5 (37.2, 94.9) | 81.6 (51.3, 101.5) | 61.6 (32.8, 93.0) | < 0.001 |
| TyG index, Mean ± SD | 9.2 ± 0.8 | 8.9 ± 0.7 | 9.2 ± 0.9 | < 0.001 |
| Hemoglobin,g/dL,Mean ± SD | 11.4 ± 2.3 | 11.3 ± 2.2 | 11.4 ± 2.3 | 0.629 |
| **In the first 2 days after entering ICU** |  |  |  |  |
| Use of high-risk nephrotoxins | 841 (59.0) | 163 (53.1) | 678 (60.6) | 0.018 |
| Use of vasopressors mediactions | 568 (39.8) | 58 (18.9) | 510 (45.6) | < 0.001 |
| Use of glucocorticoids | 288 (20.2) | 44 (14.3) | 244 (21.8) | 0.004 |
| Use of diuretics | 497 (34.9) | 87 (28.3) | 410 (36.6) | 0.007 |
| Use of acetaminophen | 862 (60.4) | 208 (67.8) | 654 (58.4) | 0.003 |
| Use of albumin 5%/20% | 173 (12.1) | 15 (4.9) | 158 (14.1) | < 0.001 |
| Time to extubation ≥24 h | 721 (50.6) | 91 (29.6) | 630 (56.3) | < 0.001 |
| **Outcomes** |  |  |  |  |
| LOS in hospital, day,Median (IQR) | 11.1 (6.2, 18.8) | 8.5 (4.7, 16.5) | 11.9 (6.8, 19.6) | < 0.001 |
| LOS in ICU, day,Median (IQR) | 5.0 (2.7, 9.8) | 2.7 (1.6, 5.1) | 5.7 (3.2, 10.7) | < 0.001 |

**Abbreviations**: ***SA-AKI***,sepsis-associated acute kidney injury;***CKD***,chronic kidney disease;***CHF,***congestive heart failure;***MI***,myocardial infarction;***SOFA***,sequential organ failure assessment;***CCI***,charlson comorbidity index;***SAPS II***,simplified acute physiology score II;***eGFR***,estimated glomerular filtration rate;***TyG index***,triglyceride-glucose index;***LOS***,length of stay;***ICU***,intensive care unit;***SD***,standard deviation;***IQR***,interquartile range.

^a^ SA-AKI was defined according to KDIGO guidelines as an increase in serum creatinine (Scr) by ≥0.3 mg/dl (≥26.5μmol/l) from baseline within 48h，or urinary output is <0.5 mL/kg/h for 6 h.

**Table S2.** Univariate linear regression analysis of the factors influencing the length of hospital stay among the study population.

| **Variables** | **β (95% CI)** | ***P*-value** |
| --- | --- | --- |
| Sex:female | -0.21 (-1.72,1.31) | 0.789 |
| Age | -0.07 (-0.11,-0.03) | 0.002 |
| Insurance:Medicare | -1.77 (-4.61,1.06) | 0.219 |
| Insurance:other | 0.35 (-2.44,3.14) | 0.805 |
| Race:African American | 0.36 (-2.44,3.15) | 0.803 |
| Race:Hispanic or Latino | 4.02 (-0.55,8.58) | 0.084 |
| Race:Asian | 9.54 (4.61,14.47) | < 0.001 |
| Race:Unknown or other | -0.15 (-1.87,1.57) | 0.866 |
| SOFA | 0.71 (0.41,1.02) | < 0.001 |
| SAPS II | 0.04 (0,0.09) | 0.078 |
| CCI | -0.11 (-0.35,0.14) | 0.386 |
| Malignant cancer | 2.76 (0.26,5.26) | 0.031 |
| CKD | -0.83 (-2.78,1.13) | 0.408 |
| Liver disease | 1.43 (-0.45,3.3) | 0.136 |
| Diabetes | 0 (-1.66,1.66) | 0.998 |
| Chronic pulmonary disease | -0.61 (-2.38,1.15) | 0.495 |
| MI | -3.22 (-5.05,-1.39) | < 0.001 |
| CHF | -1.45 (-3.1,0.21) | 0.087 |
| Hemoglobin | -0.3 (-0.64,0.03) | 0.072 |
| TyG index | 1.76 (0.88,2.65) | < 0.001 |
| eGFR | 0 (-0.02,0.02) | 0.818 |
| Serum creatinine | 0.35 (-0.12,0.83) | 0.146 |
| Use of high-risk nephrotoxins | 0.51 (-1.02,2.03) | 0.515 |
| Use of diuretics | 0.12 (-1.45,1.7) | 0.877 |
| Use of glucocorticoids | 1.49 (-0.37,3.36) | 0.117 |
| Use of vasopressors mediactions | 1.53 (0,3.07) | 0.049 |
| Use of acetaminophen | 0.86 (-0.68,2.39) | 0.272 |
| Use of albumin 5%/20% | 4.4 (2.11,6.68) | < 0.001 |
| Time to extubation ≥24 h | 2.46 (0.96,3.95) | 0.001 |

**Abbreviations**: ***CKD***,chronic kidney disease;***CHF,***congestive heart failure;***MI***,myocardial infarction;***SOFA***,sequential organ failure assessment;***CCI***,charlson comorbidity index;***SAPS II***,simplified acute physiology score II;***eGFR***,estimated glomerular filtration rate;***TyG index***,triglyceride-glucose index;***SA-AKI***,sepsis-associated acute kidney injury;***OR***,odds ratio;***CI***,confidence interval.

**Table S3.** Univariate linear regression analysis of the factors influencing the length of ICU stay among the study population.

| **Variables** | **β (95% CI)** | ***P*-value** |
| --- | --- | --- |
| Sex:female | -0.22 (-1,0.55) | 0.572 |
| Age | -0.03 (-0.05,0) | 0.017 |
| Insurance:Medicare | 0.36 (-1.09,1.82) | 0.622 |
| Insurance:other | 0.99 (-0.44,2.41) | 0.176 |
| Race:African American | 1.09 (-0.34,2.52) | 0.137 |
| Race:Hispanic or Latino | 0.33 (-2.01,2.67) | 0.78 |
| Race:Asian | 1.96 (-0.57,4.49) | 0.129 |
| Race:Unknown or other | 1.28 (0.39,2.16) | 0.005 |
| SOFA | 0.2 (0.04,0.35) | 0.014 |
| SAPS II | 0.02 (0,0.05) | 0.065 |
| CCI | -0.06 (-0.18,0.07) | 0.363 |
| Malignant cancer | -0.98 (-2.26,0.3) | 0.133 |
| CKD | -0.12 (-1.12,0.88) | 0.812 |
| Liver disease | -0.33 (-1.29,0.63) | 0.494 |
| Diabetes | 0.44 (-0.4,1.29) | 0.305 |
| Chronic pulmonary disease | 0.8 (-0.1,1.7) | 0.083 |
| MI | -1.39 (-2.32,-0.45) | 0.004 |
| CHF | -0.18 (-1.03,0.67) | 0.68 |
| Hemoglobin | 0.15 (-0.02,0.31) | 0.093 |
| TyG index | 1.36 (0.91,1.81) | < 0.001 |
| eGFR | 0 (-0.01,0.01) | 0.586 |
| Serum creatinine | 0.12 (-0.13,0.36) | 0.345 |
| Use of high-risk nephrotoxins | 0.08 (-0.7,0.86) | 0.834 |
| Use of diuretics | 0.93 (0.13,1.73) | 0.023 |
| Use of glucocorticoids | 0.31 (-0.65,1.26) | 0.528 |
| Use of vasopressors mediactions | 1.74 (0.97,2.52) | < 0.001 |
| Use of acetaminophen | 0.79 (0.01,1.58) | 0.048 |
| Use of albumin 5%/20% | 0.3 (-0.88,1.47) | 0.622 |
| Time to extubation ≥24 h | 2.2 (1.44,2.96) | < 0.001 |

**Abbreviations**: ***CKD***,chronic kidney disease;***CHF,***congestive heart failure;***MI***,myocardial infarction;***SOFA***,sequential organ failure assessment;***CCI***,charlson comorbidity index;***SAPS II***,simplified acute physiology score II;***eGFR***,estimated glomerular filtration rate;***TyG index***,triglyceride-glucose index;***SA-AKI,***sepsis-associated acute kidney injury;***OR***,odds ratio;***CI***,confidence interval.

**Table S4.** Percentage of missing data in the included variables of the study population in MIMIV-IV database.

| **Categories** | **Miss.frequency** | **Miss.percentage(%)** |
| --- | --- | --- |
| **Demographic** |  |  |
| Age | 0 | 0 |
| Sex | 0 | 0 |
| Race | 0 | 0 |
| Insurance | 0 | 0 |
| **Comorbidities** |  |  |
| Chronic pulmonary disease | 0 | 0 |
| CHF | 0 | 0 |
| Diabetes | 0 | 0 |
| Liver diseas | 0 | 0 |
| Malignant cancer | 0 | 0 |
| MI | 0 | 0 |
| CKD | 0 | 0 |
| **Severity scores** |  |  |
| CCI | 0 | 0 |
| SOFA | 0 | 0 |
| SAPSII | 0 | 0 |
| **Laboratory parameters** |  |  |
| Serum creatinine | 0 | 0 |
| eGFR | 0 | 0 |
| Hemoglobin | 2 | 0.14 |
| TyG index | 0 | 0 |
| **In the first 2 days after entering ICU** |  |  |
| Use of diuretics | 0 | 0 |
| Use of glucocorticoids | 0 | 0 |
| Use of acetaminophen | 0 | 0 |
| Use of albumin 5%/20% | 0 | 0 |
| Use of high-risk nephrotoxins | 0 | 0 |
| Use of vasopressors mediactions | 0 | 0 |
| Time to extubation ≥24 h | 0 | 0 |
| **Outcomes** |  |  |
| SA-AKI | 0 | 0 |
| LOS in hospital | 0 | 0 |
| LOS in ICU | 0 | 0 |

**Abbreviations**: ***CKD***,chronic kidney disease;***CHF,***congestive heart failure;***MI***,myocardial infarction;***SOFA***,sequential organ failure assessment;***CCI***,charlson comorbidity index;***SAPS II***,simplified acute physiology score II;***eGFR***,estimated glomerular filtration rate;***TyG index***,triglyceride-glucose index;***SA-AKI***,sepsis-associated acute kidney injury;***LOS***,length of stay;***ICU***,intensive care unit.

**Table S5.** List of medications administered in the first 2 days of ICU admission.

| **High-risk nephrotoxins** | | |  |
| --- | --- | --- | --- |
| Acyclovir | Amikacin | Amphotericin B | Cisplatin |
| Cyclophosphamide | Cyclosporine | Gentamicin | Hydroxyurea |
| Ibuprofen | Imatinib | Indomethacin | Ketorolac |
| Mercaptopurine | Mesalamine | Methotrexate | Naproxen |
| Neomycin | Sirolimus | Tacrolimus | Tobramycin |
| Vancomycin |  |  |  |
| **Diuretics** | |  |  |
| Mannitol 20% | Acetazolamide | Furosemide | Hydrochlorothiazide |
| Spironolactone |  |  |  |
| **Glucocorticoids** | | |  |
| Betamethasone | Cortisone | Dexamethasone | Hydrocortisone |
| Prednisolone | Prednisone | Triamcinolone | Methylprednisolone |
| **Vasopressors** | | |  |
| Dopamine | Epinephrine | Norepinephrine | Phenylephrine |
| Vasopressin | Dobutamine |  |  |
| **Others** | |  |  |
| Acetaminophen | Albumin 5/25% |  |  |
